# Transoral Robotic Surgery in the HPV Era

**DOI:** 10.5041/RMMJ.10144

**Published:** 2014-04-28

**Authors:** Irit Duek, Salem Billan, Moran Amit, Ziv Gil

**Affiliations:** 1 Department of Otolaryngology Head and Neck Surgery, Rambam Health Care Campus, Haifa, Israel;; 2 The Oncology Institute, Rambam Health Care Campus, Haifa, Israel;; 3 The Laboratory for Applied Cancer Research, the Clinical Research Institute, Rambam Health Care Campus, The Technion, Haifa, Israel

**Keywords:** da Vinci^®^ Surgical System, human papillomavirus, minimally invasive, oropharyngeal squamous cell carcinoma, transoral robotic surgery

## Abstract

The incidence of oropharyngeal squamous cell carcinoma (OPSCC) has markedly increased over the last three decades mostly due to human papillomavirus (HPV)-related infections. Cancers resulting from HPV infection bear a better prognosis than those that are smoking-related. Because HPV-positive patients are often younger, with lower rates of co-morbid illness and longer overall life expectancies, long-term sequelae of therapy have become an important issue. Treatment of oropharyngeal cancers has typically involved the use of radiation and chemotherapy to avoid the morbidity of open surgery which included mandibulotomy and composite resection. Transoral robotic surgery (TORS) is an emerging treatment option for this disease, avoiding the morbidity of open approaches while providing excellent oncologic and functional outcomes. With overall survival rate at 2 years exceeding 80%, and local failure rate of less than 3%, patients receiving TORS report relatively good health-related quality of life (QOL) scores. The aim of the current review is to provide a summary of the current literature with regard to the oncologic and functional outcomes following treatment of OPSCC with TORS.

## INTRODUCTION

The rate of head and neck squamous cell carcinoma (HNSCC) has been increasing recently secondary to an epidemic of human papillomavirus (HPV)-related oropharyngeal squamous cell carcinoma (OPSCC).^1^–^6^ HPV-positive OPSCC has a unique demographic, risk factor profile, and tumor biology. The incidence of tonsil and base of tongue cancers is approximately 2 per 100,000 with an incidence increase of 3.9% and 2.1% per year, respectively.^3^,^7^ These trends have been associated with a shift in demographics to a younger population that is typically high-functioning with lower rates of co-morbid illness, with minimal or no history of tobacco use, and longer overall life expectancies.^8^–^12^ Infection with oncogenic HPV types per se is considered an independent risk factor, with an increased likelihood of over 200-fold for the development of oropharyngeal cancer.^13^–^16^ This etiologic agent may play a synergistic role in the development of oropharyngeal cancer with tobacco and alcohol use.^17^,^18^ Moreover, HPV tumor status was shown to be a strong prognostic factor for OPSCC.^19^ HPV-positive tumor status significantly improves survival, regardless of the treatment modality, compared to HPV-negative tumor status, in patients with smoking- and alcohol-related head and neck cancers.^1^,^14^,^20^ The mechanisms that underlie the improved prognosis conferred by HPV-positive disease are unknown, but are thought to be partly because of better therapeutic response to induction chemotherapy and to chemoradiation treatment.^21^–^26^ These studies focused our attention on the need to reduce treatment-related toxicity in order to improve short- and long-term quality of life (QOL) of patients.

Traditional treatments for OPSCC include surgical therapy, intensity-modulated radiation therapy (IMRT), combined chemotherapy and radiation therapy (CRT), and combinations of these modalities.^27^–^31^

Surgical approaches to the oropharynx traditionally involved skin incisions and mandibulotomy.^32^ Although this approach was effective at obtaining tumor control, the speech, swallowing, and cosmetic outcomes were poor, with a high rate of complications. In 2002, Parsons et al.^32^ analyzed the largest series reporting on the traditional approaches of treating OPSCC from 1970 to 2000. They compared outcomes of surgery versus radiation for oropharyngeal cancer and found that the 5-year cause-specific survival with surgery averaged 57%, whereas the severe complication rate was 23%. They concluded that given the higher complication rate with surgery, most oropharyngeal cancers should be treated with radiation. In the last few decades, organ preservation modalities have become the mainstay of treatment. Thus, despite excellent local control rates with primary surgery, the trend shifted towards CRT as the primary treatment for oropharyngeal carcinomas, with surgery reserved for salvage.^27^,^28^,^32^–^36^ Indeed, between 1985 and 2001, the use of definitive chemoradiotherapy for advanced oropharyngeal cancer doubled.^28^ Nonetheless, chemoradiotherapy bears considerable acute and late toxicities, such as dysphagia, mucositis, xerostomia, fibrosis, osteoradionecrosis, trismus, neutropenia, neurotoxicity, nephrotoxicity, and ototoxicity.^34^,^37^,^38^ The addition of chemotherapy to radiation therapy (RT) increases the risk of long-term gastrostomy tube dependence from 1% to 13%.^37^ These toxicity risks, and the consequent reduction in QOL, together with the combination of good prognosis and younger age of HPV-positive HNSCC, have led to increasing interest in reduction of treatment-related morbidity, in order to improve functional QOL.

There are different possibilities to decrease toxicity, one of which is the reduction of the standard dose of definitive RT. Another strategy is the replacement of cisplatin with cetuximab, a monoclonal antibody against epidermal growth factor receptor (EGFR) for chemoradiation. Cisplatin is still considered the gold standard for chemoradiation, but cetuximab may be less toxic with comparable treatment results in retrospective analyses.^39^–^41^ Another strategy to reduce morbidity for HPV-positive patients is the primary treatment by surgery employing new, minimally invasive surgical approaches that allow resection of OPSCC via an oral approach, especially transoral robotic surgery (TORS).

Effective primary surgical management may provide an opportunity for deintensification of adjuvant treatments with resultant improvements in patients’ post-treatment QOL, without compromising oncologic outcomes. The ability to avoid incisions in the face and neck preserves neuromuscular structures that are critical for speech and swallowing. Preliminary case series of TORS have reported encouraging oncologic, functional, and QOL outcomes compared with primary CRT.^42^,^43^ TORS has been used for OPSCC treatment for several years driven by the desire to offer a less morbid alternative to chemoradiation, so long-term functional and oncologic results are increasingly available to allow comparison of this technique with traditional approaches.^29^,^30^,^44^–^46^

The aim of the current review is to provide an evaluation of the existing literature with regard to the oncologic and functional outcomes following treatment of OPSCC with TORS.

## TECHNICAL ADVANTAGES OF TORS

Transoral robotic surgery was first introduced into the literature by Weinstein et al.^47^ in 2005 with their case report of a supraglottic laryngectomy performed in a canine model. The development of TORS in its various human applications has been steadily progressing since, with feasibility studies confirming the safety and usability of this technology in human patients.^48^,^49^ TORS was approved by the United States Food and Drug Administration (FDA) in December of 2009 for treatment of head and neck malignancies.

TORS has several technical advantages; first, translation of the surgeon’s hand to scaled down movements of the robotic arms, filters tremors. This feature provides more accurate dissection in tenuous areas such as over the internal carotid artery in parapharyngeal dissections. Second, the three-dimensional high-definition image at the surgeon’s console provides improved visualization, which helps to compensate the lack of haptic feedback.^50^,^51^ Third, angled scopes also improve visualization and help the surgeon navigate around corners, as is often needed in tongue base surgery.^48^,^49^,^52^,^53^ Fourth, the articulated robotic arms add degrees of freedom to surgical movements. Fifth, studies have shown that robotic surgery has a more favorable learning curve than traditional laparoscopic/endoscopic and open surgery.^51^

Given the benefit of infield optics provided by the robot-mounted binocular endoscope, and the two low-profile articulating arms that can be placed in the oropharynx while the surgeon sits at a separate console to control the instruments, visualization and access challenges associated with more traditional transoral techniques are overcome with the use of TORS.^50^

Moreover, with improved visualization and freedom of motion, TORS allows excellent access to the oropharyngeal sub-sites, making it useful not only for ablative purposes, but also potentially as a diagnostic modality.^54^

TORS has been used to treat variable tumors at variable sites in the head and neck region, such as the oral cavity, pharynx (oropharynx, hypopharynx), parapharyngeal space, and larynx.^30^,^42^,^46^,^55^ At the oropharynx (tonsils, base of tongue, soft palate), TORS has been used to treat variable tumors, such as squamous cell carcinoma, adenoid cystic carcinoma, mucoepidermoid carcinoma, and neuroendocrine carcinoma.^56^

## TORS FEASIBILITY

Hockstein et al.^48^ demonstrated that several surgical procedures including a tongue base resection were technically feasible using the da Vinci^®^ Surgical System (Intuitive Surgical, Inc., Sunnyvale, CA, USA). Operative set-up times have been reported to be between 2 minutes and 140 minutes. Generally, average set-up times after preliminary experience within the TORS team are under 30 minutes. O’Malley et al.^52^ described the first series of TORS tongue base resections for squamous cell carcinoma (SCC). The set-up time ranged between 40 and 52 minutes in three cases, and the majority was in positioning the patient. The learning curve for surgeons carrying out TORS resections has been demonstrated to be short for early-stage cases, likely fewer than 10 cases, with improvements in operative time (but not in oncologic outcomes) evident as learning occurs.^30^,^52^,^55^

## ONCOLOGIC OUTCOMES

The oncologic outcomes from TORS surgery for oropharyngeal cancer seem promising (Tables 1, 2, and 3).^20^,^56^–^63^ TORS as a primary surgical modality, followed by adjuvant therapy as indicated, offers disease control in both HPV-negative and HPV-positive patients.^20^ Weinstein et al.^60^ showed that even as the only modality used for treatment of pathologically low-risk OPSCCs, TORS provides high local control and is associated with low surgical morbidity. The value of TORS was shown also as an alternative surgical approach to recurrent tumors of the oropharynx with acceptable oncologic outcomes and better functional outcomes than traditional open surgical approaches.^64^

**Table 1.** Characteristics of TORS Studies Included in the Review

**TORS Study**	**Year**	**Number of Patients**	**Follow-up (Months)**	**Final Negative Margins**	**HPV Status**	**Adjuvant Treatment**		
						**None**	**RT Alone**	**CRT**
Cohen et al.^20^	2011	50	24	47 (94%)	HPV-positive: 37/50 (74%)	9 (18%)	12 (24%)^*^	27 (54%)^*^
Weinstein et al.^53^	2010	31	24	31 (100%)		7 (22%)	12 (39%)	12 (39%)
Park et al.^56^	2013	39 oropharyngeal carcinoma of various types (32 OPSCC)	24	37 (95%)		14 (35.9%)	21 (53.85%)	4 (10.25%)
Cognetti et al.^58^	2012	30	18	29 (97%)		8 (26%)	11 (37%)	11 (37%)
Hurtuk et al.^59^	2011	54	11.8	50 (92.6%)		5 (9%)	15 (28%)	34 (63%)
Weinstein et al.^61^	2010	47	26	46 (98%)		5 (10.6%)	18 (38.3%)	24 (51%)
Genden et al.^62^	2011	30	18	30 (100%)		5 (17%)	11 (36%)	14 (47%)
White et al.^63^	2010	89 HNSCC of all sub-sites (77 OPSCC)	24	89 (100%)		33 (37%)	13 (15%)	43 (48%)
Moore et al.^68^	2012	66	36	65 (98.5%)	HPV-positive: 44/61 (72.1%)	11 (16.7%)	14 (21.2)	41 (62.1%)

* 2 more patients (4%) received adjuvant chemotherapy.

CRT chemotherapy and radiation therapy; HNSCC, head and neck squamous cell carcinoma; HPV human papillomavirus; OPSCC, oropharyngeal squamous cell carcinoma; RT, radiation therapy; TORS, transoral robotic surgery.

**Table 2.** Survival Outcomes Following TORS for OPSCC.

**TORS Study**	**2-Year Disease-specific Survival**	**2-Year Overall Survival**
Cohen et al.^20^	25/27 (92.6%)	25/31 (80.6%)
Park et al.^56^		31/32 (96%)
Cognetti et al.^58^	28 (93.3%)	28 (93.3%)
Weinstein et al.^61^	27/30 (90%)	27/33 (82%)
Genden et al.^62^		27 (90%)
White et al.^63^		Primary TORS cohort (89.3%)
Moore et al.^68^	63 (95.5%)	63 (95.5%)

OPSCC, oropharyngeal squamous cell carcinoma; TORS, transoral robotic surgery.

**Table 3.** Patterns of Failure Outcomes Following TORS for OPSCC.

**TORS Study**	**Follow-up (Months)**	Disease-Free Survival^*^		Control^*^		

				**Local**	**Regional**	**Distant**

Cohen et al.^20^	24	24/27 (89%)		27/27 (100%)	26/27 (96.3%)	24/27 (89%)
Weinstein et al.^53^	24	30 (97%)		31 (10%)	30 (97%)	30 (97%)
Park et al.^56^	24	29 (92%)				
Cognetti et al.^58^	18	1 year: 27 (90%)		29 (97%)	29 (97%)	27 (90%)
Hurtuk et al.^59^	11.8	52 (96.3%)		53 (98.15%)		
Weinstein et al.^61^	26	1 year:45/47 (96%)	2 years: 26/33 (79%)	46 (98%)	45 (96%)	43 (91.5%)
Genden et al.^62^	18	23 (78%)		27 (91%)		28 (93%)
White et al.^63^	24	77/89 (86.5%)		79 (89%)		88 (99%)
Moore et al.^68^	36	61 (92.4%)		64 (97%)	62 (94%)	65 (98.4%)

* Corresponding to follow-up time, unless otherwise mentioned.

OPSCC, oropharyngeal squamous cell carcinoma; TORS, transoral robotic surgery.

### Margin Status and Disease Control

Achieving negative margins intraoperatively has been demonstrated to be an important prognostic factor in transoral surgery for OPSCC.^65^,^66^ Haughey et al.^67^ found that the presence of a positive margin after surgery in 7% of their patients raised the risk of death 2.5-fold to 3.0-fold compared with that for patients with negative margins. With TORS, it is relatively easy and less morbid to achieve 5 mm clear surgical margins around a multiplanar en bloc resection in the area of interest, especially in the oropharynx, without requiring mandible split or floor of mouth release. Moore et al.^68^ presented in their study 66 consecutive patients who underwent TORS as the primary treatment for OPSCC and were followed up for a minimum of 2 years. In their series, margins were cleared in 65 of the 66 patients at the time of primary surgery, and 3-year recurrence-free survival was achieved in 92.4% of the patients. In the setting of node-negative disease with no primary site adverse features, the risk of local-regional relapse with observation was less than 10%.^69^ Weinstein et al.^60^ suggested that TORS provides accurate pathologic evaluation when the surgeon verifies clear orientation of the specimen.

The high rate of negative margins following TORS has implications for the design and dosing of adjuvant radiotherapy to the primary site. The possible reduction of dose in adjuvant radiotherapy is a combination of the reliable margin status achieved following TORS and the inherent better prognosis of HPV-related SCC.^53^,^70^ The role of postoperative radiation in patients with HPV-positive or negative OPSCC is a subject of ongoing research. Reduction of radiation dose and sparing of chemotherapy has the potential of reducing morbidity and improving short- and long-term QOL.^60^,^71^,^72^ Six reports demonstrated that 8%–37% of patients were spared radiation and 48%–74% of patients did not require chemotherapy after TORS.^20^,^53^,^59^,^62^,^63^,^72^ This selective approach has the potential to reduce toxicity and the risk of late complications and reserve treatment modalities for second primary tumors or recurrences.^37^

### Local Control, Disease-specific Survival, and Overall Survival

Clinical trials reporting the results of chemoradiation treatment for OPSCC report 3-year disease-free survival and overall survival rates of 42% to 76.5% and 51% to 85%, respectively.^27^,^73^ Preliminary data relating to local control, disease-specific survival, and overall survival using upfront TORS are encouraging, with overall survival rates at 1 year exceeding 90% and with 2-year survival rates >80%.^20^,^61^,^63^ Small series reported local failure rates for TORS between 0% and 3% with median follow-up rates ranging from 18 months to 2 years.^20^,^61^,^63^ Regional recurrence rates varied between 2% and 8%,^20^,^61^,^63^ while distant disease was reported in 1%–9% of patients.^20^,^61^–^63^ Nonetheless, a factor confounding interpretation of the true effectiveness of TORS on local control has been the use of postoperative radiation or chemoradiation therapy due to positive nodal metastases or extra-capsular spread.^61^ In one large series, for example, only 2% of patients had positive margins, but the presence of nodal metastases resulted in 85% of patients receiving postoperative radiation to the neck and primary site with or without chemotherapy.^61^ The use of radiotherapy as well as chemotherapy in a large number of cases calls into question whether the success at the primary site was related to the surgical procedure itself. In an effort to discover whether TORS alone, without postoperative radiation or chemoradiation therapy, can provide effective local control for mucosal OPSCC, Weinstein et al.^60^ studied a cohort of patients from two consecutive TORS single-arm, prospective, observational trials performed at the University of Pennsylvania. Within both of these studies was a cohort of patients with previously untreated OPSCC who underwent TORS alone. The primary objective of their study was to assess the local control rate for a series of patients with OPSCC who were treated with TORS followed by staged neck dissection as indicated without postoperative radiation therapy or chemotherapy. Secondary end-points included evaluation of the safety and efficacy of this approach. In their prospective, single-center, observational study, Weinstein et al.^60^ tried to evaluate local control following TORS with the da Vinci^®^ Surgical System as a single treatment modality for OPSCC. Thirty patients were enrolled with previously untreated OPSCC and no prior head and neck radiation therapy. Follow-up duration was at least 18 months. Final pathologic evaluation revealed 10 cases (33%) that were pathologic node-positive. Only 1 patient (3%) had a positive margin after primary resection; further resection achieved a final negative margin, thus avoiding the morbidity associated with chemoradiation therapy. Perineural invasion was noted in 3 tumors (10%). No patient received postoperative adjuvant therapy. At a mean follow-up of 2.7 years (range, 1.5–5.1 years), there was 1 patient with local failure (3%). Surprisingly, 16 of 30 patients had overall clinical stage 3 or 4 disease (53%) and had no local failures at the primary site despite the lack of adjuvant of therapy. Under the treatment regimen of primary TORS and staged neck dissection without postoperative radiation, this cohort achieved local, regional, and distant disease control in 29 of 30 (97%), 27 of 30 (90%), and 30 of 30 (100%) cases, respectively, at a minimum follow-up of 18 months. Overall survival for this cohort at the time of last follow-up was 30 of 30 (100%), also at a minimum follow-up of 18 months. The findings of this study confirmed the findings of prior studies that the morbidity of TORS alone for oropharyngeal cancer is low because there was no requirement for permanent feeding tubes and no perioperative mortality.^60^ The authors concluded that, as the only modality used for treatment of pathologically low-risk OPSCCs, TORS provides high local control and is associated with low surgical morbidity.^60^

## FUNCTIONAL OUTCOMES (TABLE 4)

In the wake of the HPV oropharyngeal cancer epidemic in the recent years, it is imperative to have treatment strategies that optimize post-treatment QOL for these patients. Initial, limited QOL data have shown that speech, eating, social, and overall QOL domains tend to decrease from baseline but remain high at 3 months post TORS.^74^–^78^

**Table 4.** Functional Outcomes Following TORS for OPSCC—Short-and Long-term.

**TORS Study**	**Number of Patients**	**Tracheostomy Dependency Rates**		**Gastrostomy Tube Dependency Rates**		
	
		**Short-term**	**1 Year**	**Short-term**	**1 Year**	**2 Years**

Moore et al.^29^	102	13.7%	0.98%	15.6%		3.92%
Moore et al.^30^	45	31%	0%	18%	0%	0%
Weinstein et al.^53^	31				2.40%	0%
Cognetti et al.^58^	30	0%	0%	9%	7%	
Hurtuk et al.^59^	54	0%–31%	0%		7.50%	
Weinstein et al.^60^	30	3.33%	0%		0%	0%
Genden et al.^62^	30		0%		0%	0%
White et al.^63^	89 HNSCC of all sub-sites (77 OPSCC)					0%
Moore et al.^68^	66	25.80%	1.50%	3%	27.30%	4.50%
Iseli et al.^72^	54	0%	0%	17%	17%	
Genden et al.^76^	20	0%	0%	0%	0%	0%

OPSCC, oropharyngeal squamous cell carcinoma; TORS, transoral robotic surgery.

TORS facilitates surgical access to the lower sub-sites of the upper aerodigestive tract without the need for traditional methods requiring open surgical approaches. As such, it is an approach to preserve the organ and maximize function.^30^,^42^,^45^,^61^ The impact of TORS on airway control and swallowing function is considered less than the impact of open surgical approaches, which frequently require tracheostomy and feeding tube placement. In conventional open surgery, the lesion is widely resected, and the sites are usually reconstructed with a free flap. However, anatomical reconstruction with a free flap does not necessarily result in the functional restoration of organs. It could also injure important structures involved in swallowing, including the muscles of the floor of the mouth and the constrictor muscle, which would lead to impaired swallowing. Park et al.^56^ evaluated prospectively the functional outcomes of patients treated with TORS in comparison with patients treated conventionally with transoral approach or mandibulotomy during the same period of the study. There was a significant difference in swallowing, time to decannulation, and hospitalization period between the two groups. In the TORS group, patients completely recovered the ability to swallow after 6 days. In contrast, patients undergoing conventional surgery did not completely recover their swallowing until 12 days. Those in the TORS group had more rapid functional recoveries of swallowing and decannulation, and had shorter hospital stays.

TORS for OPSCC also offers improved functional outcomes when compared to non-surgical treatment with radiotherapy or chemoradiotherapy.^30^,^46^,^61^–^63^,^72^,^74^–^76^ Patients receiving TORS alone report better health-related QOL compared to individuals receiving TORS and adjuvant radiation or chemoradiation.^43^,^50^,^60^ Genden et al.^62^ performed a case-control study to compare QOL between patients undergoing TORS and those undergoing primary chemoradiotherapy. Between 2007 and 2009, 30 patients with HNSCC were treated with primary TORS and adjuvant therapy as indicated. Patients were evaluated before treatment, after treatment, and at subsequent 3-month intervals after completing treatment to determine their disease and head and neck-specific functional status using the Performance Status Scale for Head and Neck Cancer and the Functional Oral Intake Score (FOIS). Functional scores were compared to a matched group of head and neck patients treated with primary CRT. TORS was associated with better short-term eating ability, better diet, and FOIS at 2 weeks after completion of treatment. In contrast to TORS patients who returned to baseline, the CRT group continued to have decreased oral intake and FOIS at 12 months.

It is well recognized that adjuvant radiation therapy and CRT can cause temporary mucositis and edema that impair swallowing function and QOL.^50^,^67^ In comparison, several studies reported low complication rates and favorable swallowing outcomes following TORS with a return-to-swallowing period of 0–14 days.^30^,^46^,^50^,^59^,^72^,^76^–^78^ Nevertheless, it is expected that objective swallowing ability of these patients will deteriorate with adjuvant treatment.^43^,^50^,^67^,^68^,^78^,^79^ Furthermore, radiation therapy may cause irreversible long-term fibrosis and impaired mobility of the upper aerodigestive tract,^50^ which can result in poor long-term functional recovery.^43^

A retrospective analysis of three Radiation Therapy Oncology Group (RTOG) trials suggested that the rate of severe late toxicities in patients receiving chemoradiotherapy is 35% for patients with oropharyngeal cancer.^37^ Long-term percutaneous endoscopic gastrostomy (PEG) tube dependency is often used as a marker of treatment-related late toxicity. Favorable gastrostomy tube rates (0%–9.5% at 1 year and 0% at 2 years post treatment) have been reported following TORS, compared to 9%–39% at 1 year in patients receiving CRT(Table 4).^27^,^30^,^42^,^61^,^62^,^72^–^74^ Swallowing-related QOL is reported to decrease immediately following TORS, but has been demonstrated to improve by 1 year post treatment, with possible further improvement thereafter.^79^ In the study of Cognetti et al.,^58^ most patients resumed oral intake by postoperative day 1, with 91% of patients tolerating oral intake at the first postoperative visit. In the 12 patients who were taking an oral diet with tube feed supplementation, the PEG tube had been placed for anticipated adjuvant therapy with chemoradiation based on clinical staging. In those patients with at least 12 months’ follow-up, two continued to have a PEG tube. The rate of 7% PEG dependence is consistent with previously published data from the pioneering TORS centers (0%–17% PEG dependence).^20^,^53^,^58^,^59^,^62^,^63^,^72^ Moore et al.^68^ showed that, even after complete TORS resection of bulky tumors, swallowing function that is impaired in the immediate postoperative period improves during the first several weeks of healing. Swallowing function dropped during adjuvant therapy, and 27.3% of patients required gastrostomy tube placement to complete adjuvant therapy. Despite the temporary decrease in swallowing function, swallowing function improved over time; ultimately, 95.5% of the patients were able to maintain their nutrition by an oral diet.^68^ Dziegielewski et al.^50^ evaluated the functional outcomes of 81 patients with previously untreated OPSCC who underwent TORS using the Head and Neck Cancer Inventory (HNCI), at 3 weeks and 3, 6, and 12 months postoperatively. There were overall declines in speech, eating, aesthetic, social, and overall QOL domains in the early postoperative periods, 3 weeks after TORS. All health-related QOL scores continued to drop and bottomed out at 3 months post TORS. This time frame coincides with RT and/or CRT treatment, during which patients experience acute toxic effects of adjuvant treatment.^43^,^50^,^78^ However, at 1 year post TORS, scores for aesthetic, social, and overall QOL remained high. Most patients experiencing RT and/or CRT disturbances tend to recover by 12 months, and their scores return to intermediate to high levels. Radiation therapy was negatively correlated with multiple QOL domains, and age older than 55 years correlated with lower speech and aesthetic scores. HPV status did not correlate with any QOL domain. Patients who avoided any adjuvant treatment showed superior QOL, as supported by other data.^43^,^78^,^79^ All patients in the Dziegielewski et al.^50^ study were able to tolerate a full oral diet by the time of hospital discharge. One-fifth of patients required a gastrostomy tube at some point after TORS, with 24% still using their gastrostomy tube at 6 months and 9%at 12 months. Greater extent of TORS (>1 oropharyngeal site resected) and age older than 55 years predicted the need for a gastrostomy tube at any point after TORS. If TORS resection included more than oneoropharyngeal sub-site, patients had a 5.6-fold increased risk of needing a gastrostomy tube. Older patients (≥55 years) were nearly five times as likely to need a gastrostomy tube after TORS compared with their younger counterparts. This is potentially owing to a lower baseline functional status and less of a capacity for aggressive swallowing therapy in the elderly. The most common indication for tube feeding was dysphagia during RT and/or CRT. A factor that predicted the need for a permanent gastrostomy tube after TORS is high T classification. Patients with T3 or T4 tumors were 27 times as likely to not be weaned from gastrostomy tube feedings. Previous TORS studies have also shown advanced T classification to be predictive of poor swallowing function and retained gastrostomy tubes.^50^,^72^

Although most authors were using perioperative tracheostomy tubes with the introduction of TORS, this seems to be a passing trend. In the study of Cognetti et al.,^58^ most patients (76%) were extubated at the conclusion of TORS. The location of the tumor resection affected the likelihood of intubation postoperatively. Only 3/21 (14.3%) tonsillar resections remained intubated, whereas 7/22 (31.8%) base of tongue resections remained intubated. All intubated patients were extubated within 36 hours, with the majority being extubated the first morning post operation. The current literature reports tracheostomy rates of 0% to 31%, with most authors demonstrating the safety of the technique without a surgical airway.^50^

## ADVERSE EVENTS

A recent systematic review^80^ compared the effectiveness of TORS to IMRT for early T-stage oropharyngeal cancer, suggesting that survival estimates are similar between the two modalities and the differences lie in adverse events. Twenty case series including eight IMRT studies (1,287 patients) and 12 TORS studies (772 patients) were included. Patients receiving definitive IMRT also received chemotherapy (43%) or neck dissections for persistent disease (30%), whereas patients receiving TORS required adjuvant radiotherapy (26%) or chemoradiotherapy (41%). Two-year overall survival estimates ranged from 84% to 96% for IMRT and from 82% to 94% for TORS. Adverse events for IMRT included esophageal stenosis (4.8%), osteoradionecrosis (2.6%), and gastrostomy tubes (43%), and for TORS included hemorrhage (2.4%), fistula (2.5%), and gastrostomy tubes at the time of surgery (1.4%) or during adjuvant treatment (30%). Tracheostomy tubes were needed in 12% of patients at the time of surgery, but most were decannulated prior to discharge.

## FURTHER RESEARCH

Comparisons of outcomes after TORS versus chemoradiotherapy across studies are hampered by differences in baseline patient populations, selection, and treatment technique. Therefore, direct comparisons across these reported functional outcomes are difficult. According to Nichols et al.^81^ all the reports about TORS till now involve prospective or retrospective single-arm case series with varying use of adjuvant therapy without adequate controls. This is in contrast to the large number of randomized controlled trials of CRT for OPSCC. Although the data described thus far would appear to favor a surgical approach, a careful review of the literature suggests that this comparison may be biased. For example, the TORS studies include a much smaller fraction of T3/T4 tumors (0%–30%) and N3 neck disease (0%–4%) compared with CRT series (31%–86% T3/T4 and 2.5%–12% N3).^27^,^42^,^73^ There are also numerous additional confounders, among them: HPV status, the socio-economic background of patients, patient selection bias, and referral center bias. Most importantly, the majority of TORS patients receive adjuvant therapy including radiation (24%) or chemoradiation (54%), making the true benefits of TORS unclear.^20^ Given the rapid treatment paradigm shift in the absence of level I evidence with the high cost of TORS, a randomized trial is critical to guide the optimal management of OPSCC. Nichols et al.^81^ suggested a randomized phase II study with the goal of comparing the QOL in patients with OPSCC (T1–2, N0–2) after TORS versus primary RT, along with a phase III trial assessing survival. Further multi-institutional studies with standardized protocol comparing surgery with RT and/or CRT are required to determine the optimal treatment for patients with OPSCC.

## CONCLUSIONS

OPSCC is an evolving cancer that affects a younger and healthier population without traditional risk factors of tobacco and alcohol use. The subgroup of HPV-positive OPSCC is being recognized as a separate entity. TORS offers a significant opportunity to impact positively on patient QOL and post-treatment function whilst retaining satisfactory oncologic control. Preliminary data relating to local control, disease-specific survival, and overall survival using upfront TORS are encouraging, with overall survival rates at 1 year exceeding 90%, and at 2 years exceeding 80%. Local failure rates for TORS are reported to be between 0% and 3%, with median follow-up rates ranging from 18 months to 2 years.^20^,^61^,^63^ Regional recurrence rates varied between 2% and 8%,^20^,^61^,^63^ while distant disease was reported in 1%–9%.^20^,^61^–^63^ Patients receiving TORS alone report better health-related quality of life (QOL) compared to individuals receiving TORS and adjuvant radiation or chemoradiation. Although initial feasibility and case series reports are encouraging, further validation through well-designed randomized control trials is required prior to widespread shifts in accepted treatment paradigms.

## Abbreviations:

CRT: chemotherapy and radiation therapy
FDA: Food and Drug Administration
EGFR: epidermal growth factor receptor
FOIS: Functional Oral Intake Score
HNSCC: head and neck squamous cell carcinoma
HNCI: Head And Neck Cancer Inventory
HPV: human papillomavirus
IMRT: intensity-modulated radiotherapy
OPSCC: oropharyngeal squamous cell carcinoma
PEG tube: percutaneous endoscopic gastrostomy tube
QOL: quality of life
RT: radiation therapy
RTOG: Radiation Therapy Oncology Group
SCC: squamous cell carcinoma
TORS: transoral robotic surgery.
